# Multi-Substrate Biofuel Cell Utilizing Glucose, Fructose and Sucrose as the Anode Fuels †

**DOI:** 10.3390/nano10081534

**Published:** 2020-08-05

**Authors:** Michał Kizling, Maciej Dzwonek, Anna Nowak, Łukasz Tymecki, Krzysztof Stolarczyk, Agnieszka Więckowska, Renata Bilewicz

**Affiliations:** Faculty of Chemistry, University of Warsaw, 1 Pasteura Str., 02-093 Warsaw, Poland; mkizling@gmail.com (M.K.); mdzwonek@chem.uw.edu.pl (M.D.); annowak@chem.uw.edu.pl (A.N.); luktym@chem.uw.edu.pl (Ł.T.); kstolar@chem.uw.edu.pl (K.S.); awiec@chem.uw.edu.pl (A.W.)

**Keywords:** bioelectrocatalysis, cascade enzymatic electrodes, enzymatic fuel cell, nanocellulose, polypyrrole

## Abstract

A significant problem still exists with the low power output and durability of the bioelectrochemical fuel cells. We constructed a fuel cell with an enzymatic cascade at the anode for efficient energy conversion. The construction involved fabrication of the flow-through cell by three-dimensional printing. Gold nanoparticles with covalently bound naphthoquinone moieties deposited on cellulose/polypyrrole (CPPy) paper allowed us to significantly improve the catalysis rate, both at the anode and cathode of the fuel cell. The enzymatic cascade on the anode consisted of invertase, mutarotase, Flavine Adenine Dinucleotide (FAD)-dependent glucose dehydrogenase and fructose dehydrogenase. The multi-substrate anode utilized glucose, fructose, sucrose, or a combination of them, as the anode fuel and molecular oxygen were the oxidant at the laccase-based cathode. Laccase was adsorbed on the same type of naphthoquinone modified gold nanoparticles. Interestingly, the naphthoquinone modified gold nanoparticles acted as the enzyme orienting units and not as mediators since the catalyzed oxygen reduction occurred at the potential where direct electron transfer takes place. Thanks to the good catalytic and capacitive properties of the modified electrodes, the power density of the sucrose/oxygen enzymatic fuel cells (EFC) reached 0.81 mW cm^−2^, which is beneficial for a cell composed of a single cathode and anode.

## 1. Introduction

Enzymatic fuel cells (EFCs) are energy converting devices that are capable of delivering power when operating in biological solutions. High substrate specificity allows catalytic transformation even in the complex matrix solutions. EFCs have the capability to operate at room temperature and neutral pH, conditions which cannot typically be employed with a conventional fuel cell. However, the obtained power and energy densities are always significantly lower than the theoretical values, due to the fact that energy is harvested from the first oxidation step of a single enzyme. So far, most of the studies on EFCs have focused on single enzyme electrodes generating current via an enzymatically catalyzed transformation step [1,2,3]. In order to further exploit the energy stored in the substrate or broaden the range of applicable fuels, the electrodes can be modified with several enzymes working in parallel or showing cascading functions. Linear series cascades include the oxidation of ethanol to acetate [4], sucrose to gluconolactone [5], and trehalose to gluconolactone [6]. Interestingly, one of the most effective coulombic efficiencies was reported by Zhang et al., who employed the pentose phosphate pathway and obtained current density, 6 mA cm^−2^, and power density, 0.8 mW cm^−2^ [7,8]. However, most metabolic pathways are not linear, i.e., the citric acid cycle or Krebs cycle is a cyclic metabolic pathway with a multitude of different substrates along the cycle that can be oxidized, including: pyruvate [9], lactate [10], or ethanol [11]. It is important to note, however, that a linear cascade may be employed to generate the intermediates that can in turn enter a cyclic metabolic cascade. The three-enzyme cascade oxidation of sucrose uses invertase first to hydrolyze the disaccharide sucrose into two monosaccharides (glucose and fructose) and then glucose oxidase and fructose dehydrogenase to simultaneously oxidize the monosaccharides in parallel rather than in series. This cascade was limited by the slow autorotation of the glucose produced from sucrose before it was utilized by glucose oxidase [5]. Therefore, the cascade was recently improved by using the three enzymes and adding mutarotase to easily transform the glucose into the proper form for glucose oxidase [12]. Such cascades are employed more frequently for complex biofuels, where a compound is transformed into simpler molecules that react along parallel oxidation paths.

Providing good contact between the active enzyme center and the electrode surface is very important for the performance of the catalytic electrodes, therefore, systems that allow direct electron transport (DET) are the subject of continuous interest. Various nanostructures are proposed for the construction of the electrode to obtain the appropriate orientation of the enzyme relative to the electrode surface and to decrease the distance of electron transport. In the large group of possible nanostructures, platinum and gold nanoparticles, are often employed due to their attractive structural, mechanical and electronic properties [13,14,15,16]. Efficient direct electron transfer (DET) has been demonstrated using porous gold electrodes [17,18,19]. Further improvement of the performance of the electrode modified with FAD-dependent glucose dehydrogenase (GDH) was reported by Kano and coworkers when the gold porous electrode contained enzymatically implanted platinum nanoclusters (PtNCs) [20].

We have shown recently that reducing the size of gold nanoparticles below 2 nm may cause a change in the electron transport mechanism between the enzyme active site and the electrode surface from DET to mediated electron transfer (MET). Gold clusters with diameters smaller than 2 nm showed molecule-like behavior, which was manifested by the appearance of oxidation and reduction peaks separated as predicted by the HOMO-LUMO gap. The nanoparticle redox activity was used to switch the electron transport mechanism from DET in fructose dehydrogenase to MET via gold clusters and led to the decrease in fructose oxidation overpotential together with the increase in catalytic reaction rate constant [21].

A significant problem is still connected to the EFC power output and the durability of the enzymatic systems in time. While EFCs, theoretically, should be capable of exhibiting high power and densities when using biological fuels at pH close to neutral, the actual power and energy densities are generally much lower [22]. It is therefore clear that efficient energy conversion using EFCs still remains a challenge.

Xiao and coworkers reviewed recent attempts in the area of biofuel cells aiming to improve the operation of EFC so that they can serve for practical applications, e.g., achieve higher energy densities by using the enzyme cascade to completely oxidize fuels, increase power density by increasing enzyme activity, and use nanomaterials to facilitate electron transfer. EFC connections with a supercapacitor which have the perspective of practical applications were also discussed [23].

We present here the construction of EFC with an enzymatic cascade at the anode for efficient energy conversion. The construction involves fabrication of the flow-through cell by three-dimensional printing. Fabrication of the flow-through cells and other components of flow systems by means of rapid prototyping technology and, in particular, three-dimensional printing is gaining rising popularity, due to the unconventional freedom in design [24,25]. Moreover, the application of gold nanoparticles with covalently bound naphthoquinone moieties deposited on cellulose/polypyrrole (CPPy) paper as the electrode substrate allowed us to significantly improve the catalysis rate, both of the anode and cathode reactions. The maximum power density of the presented sucrose/oxygen EFC was 0.81 mW cm^−2^.

## 2. Materials and Methods

### 2.1. Materials and Chemicals

The cellulose/polypyrrole paper-based EFC was composed of an anode comprising four enzymes: invertase, mutarotase, fructose dehydrogenase and glucose dehydrogenase (FAD dependent) and a cathode based on laccase. Invertase, INV, from *Saccharomyces cerevisiae* (EC 3.2.1.26) and laccase, LAC, from *Trametes Hirsuta* (1.10.3.2) from Sigma-Aldrich (Poznan, Poland), FAD-dependent glucose dehydrogenase, GDH, (E.C. 1.1.99.10, *Aspergillus* sp.) from Sekisui Diagnostics (Maidstone, UK), D-fructose dehydrogenase, FDH, from *Gluconobacter* sp. (EC 1.1.99.11) from Sorachim, mutarotase, MUT, from porcine (EC 5.1.3.3) from Sinus Biochemistry and Electrophoresis, and GmbH were used without any further purification. Citric acid (C_6_H_8_O_7_), disodium hydrogen phosphate (Na_2_HPO_4_), ethanol (C_2_H_5_OH), glucose (C_6_H_12_O_6_), sucrose (C_12_H_22_O_11_) and fructose (C_6_H_12_O_6_) were purchased from Avantor (Gliwice, Poland). An Invertase Activity Assay Kit was purchased from Sigma-Aldrich. All other not directly specified reagents and solvents or chemicals used in the AuNPs and naphthoquinone derivative syntheses were obtained from Sigma-Aldrich (Poznan, Poland) and used without further purification. The argon gas and oxygen utilized in the experiments were purchased from Air Products (Warsaw, Poland) while the water was distilled and passed through a Milli-Q purification system. Pyrrole (Merck, Darmstadt, Germany) pyrrole-2-carboxylic acid (Sigma-Aldrich) FeCl_3_·6H_2_O (BDH Prolabo), Tween-80 (Merck), 37% HCl (Merck), KMnO_4_ (Avantor), H_2_SO_4_ (Avantor), H_2_O_2_ (Avantor) and NaCl (BDH Prolabo, Fontenay-sous-Bois, France), were used as received and were mixed with deionized water to obtain the desired concentrations.

### 2.2. Synthesis of Cellulose/Polypyrrole Composite

The *Cladophora* sp. algae cellulose was obtained as described earlier [26,27]. A dispersion of cellulose was prepared by ultrasonication (VibraCell 750 W, Sonics, Newtown, CT, USA) of 300 mg cellulose in 60 mL of deionized water. Then, 1.5 mL of pyrrole and a drop of Tween-80 were dissolved in 50 mL of 0.5 M HCl and mixed with the cellulose dispersion. Exactly 12.857 g of FeCl_3_·6H_2_O was dissolved in 100 mL 0.5 M HCl. To initiate the polymerization, the FeCl_3_ solution was added dropwise to the pyrrole and cellulose mixture. The polymerization was then left to proceed for 30 min under stirring, after which the product was collected in a Büchner funnel and washed with 5 L of 0.5 M HCl followed by 1 L of 0.1 M NaCl. The collected composite (CPPy) was pressed and dried under ambient conditions. The CPPy pieces (0.79 cm^2^, circular) utilized as electrodes had a mass of approximately 25 mg each.

### 2.3. Synthesis of the Naphthoquinone Thiol Derivative

**Scheme 1:**
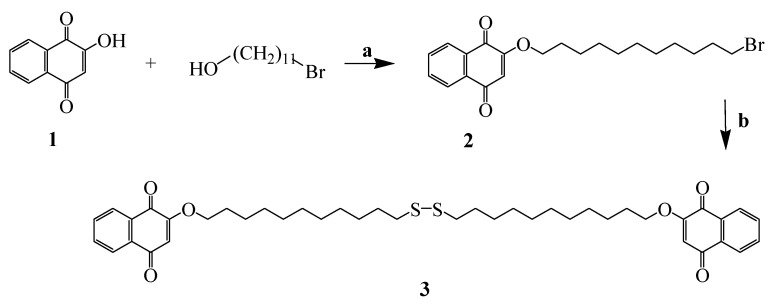
Synthesis of compound **3**. Reagents and conditions: (**a**) triphenylphosphine, diisopropyl azodicarboxylate (DIAD), tetrahydrofuran (THF), 5 °C, 70%; (**b**) hexamethyldisilathiane (HMDT), tetrabutylammonium fluoride hydrate (TBAF), tetrahydrofuran (THF), room temperature, 54%.

The commercially available 2-hydroxy-1,4-naphthoquinone served as the starting material (Scheme 1 **1**). It was converted to the corresponding bromo compound, Scheme 1 **2**, which in the reaction with hexamethyldisilathiane, carried out in the presence of tetrabutylammonium fluoride, formed the disulfide Scheme 1 **3**.

Nuclear magnetic resonance spectra were recorded in CDCl_3_ solutions using Bruker AVANCE 300 MHz (Karlsruhe, Germany) and Bruker AVANCE 400 MHz instruments. Chemical shifts (δ) were reported in parts per million relative to (CH_3_)_4_Si (δ 0.00) or solvent signal as an internal standard. Signals in 1H NMR spectra were described using the following abbreviations: s-singlet, d-doublet, t-triplet, m-multiplet. High-resolution mass spectra were recorded on Mass Quattro LC spectrometers using electrospray ionization (ESI) technique.

Reaction temperatures refer to external bath temperatures. Tetrahydrofuran was distilled from Na/benzophenone. The organic extracts were dried over anhydrous MgSO_4_, filtered and concentrated using a rotary evaporator. Reactions were monitored by thin-layer chromatography (TLC) using aluminum-backed MERCK 60 silica gel plates (Darmstadt, Germany) (0.2 mm thickness). The chromatograms were visualized first with ultraviolet light (254 nm) and then by immersion in a cerium-molybdenum solution (5 g Ce(SO_4_)_2_ × 4 H_2_O, 12 g phosphomolybdic acid, 30 mL H_2_SO_4_ and 480 mL H_2_O) followed by heating.

**2-(11′-Bromoundecyloxy) naphthalene-1,4-dione (2).** A solution of 2- hydroxynaphthalene-1,4-dione (1; 1.0 g, 5.71 mmol) in anhydrous tetrahydrofuran (THF) (80 mL) was placed in a 100-mL two-necked round-bottom flask fitted with a thermometer and a dropping funnel. To this solution was added 11-bromoundecan-1-ol (1.43 g, 5.71 mmol) and triphenylphosphine (1.64 g, 6.27 mmol). After cooling in the ice-water bath to 5 °C, diisopropyl azodicarboxylate (1.27 g, 6.27 mmol) in anhydrous tetrahydrofuran (THF) (10 mL) was added dropwise to the stirred solution at such a rate that the temperature did not rise above 10 °C. The mixture was allowed to warm to room temperature and was stirred overnight. Then the mixture was concentrated to ca. 10 mL and methanol (MeOH) added. The solution was filtered to give the yellow solid 2 (1.56 g, 70%): 1H NMR (400 MHz, CDCl_3_) δ 8.12 (1H, d, *J* = 7.0 Hz), 8.08 (1H, d, *J* = 7.0 Hz), 7.77–7.68 (2H, m), 6.15 (1H, s), 4.01 (2H, t, *J* = 6.7 Hz), 3.41 (2H, t, *J* = 6.8 Hz), 1.93–1.82 (4H, m), 1.49–1.28 (14H, m).

**2-{11′-[11″-[1‴,4‴-Dioxo-1‴,4‴-dihydro-naphthalen-2’’’-yloxy)-undecyl]-disulfanyl]-undecyloxy}-[1,4]naphthoquinone (3).** Hexamethyldisilathiane (156.1 mg, 0.84 mmol) was added to a solution of the 2-(11-bromoundecyloxy)naphthalene-1,4-dione (2; 341.4 mg, 0.84 mmol) in anhydrous tetrahydrofuran (THF) (12 mL). The mixture was stirred for 15 min at room temperature under argon and tetrabutylammonium fluoride hydrate (0.84 mL, 0.84 mmol) was added. The mixture was stirred for 1 h and it was then extracted with ethyl acetate. The organic phase was dried (MgSO_4_) and evaporated. The residue was purified by column chromatography over silica using hexane/ethyl acetate (9:1 → 8:2) as an eluent to give solid 3 (187 mg, 54%): 1H NMR (300 MHz, CDCl_3_) δ 8.13–8.07 (4H, m), 7.74–7.69 (4H, m), 6.15 (2H, s), 4.00 (4H, t, *J* = 6.1 Hz), 2.68 (4H, t, *J* = 6.2 Hz), 1.95–1.67 (8H, m), 1.52–1.26 (28H, m); HRMS (ESI) mass calculated for C_42_H_54_O_6_S_2_Na (M^+^ + Na) 741.2234, measured 741.2230.

### 2.4. Gold Nanoparticles Modified with the Naphthoquinone Derivative

First, 150 mg of HAuCl_4_·3H_2_O (0.381 mmol) was dissolved in 40 mL anhydrous THF and 1.2 eq. TOAB (250 mg, 0.457 mmol) was added. The reaction mixture was stirred and cooled in the dry ice/EtOH bath for 30 min with an argon purge. Then, an NQ derivative (0.26 eq., 0.1 mmol) in 10 mL of anhydrous THF was added dropwise. The reaction mixture was stirred for about 15 h in the dry ice/EtOH bath under Ar atmosphere and then 5.7 eq. (2.2 mmol) of 1-hexanethiol (C6) was added dropwise in 10 mL of anhydrous THF with constant stirring and cooling in the dry ice/EtOH bath overnight under Ar atmosphere. Next, the 10 eq. of NaBH_4_ (3.81 mmol, 144.1 mg) in 10 mL ice-cold H_2_O was added dropwise. Then, the reaction mixture was stirred for 4 days and the sample was filtered. The reaction was partitioned between CH_2_Cl_2_ (10 mL) and water (10 mL). The layers were separated and the combined organic layers were washed with water (3 × 10 mL), dried over anhydrous Na_2_SO_4_, filtered and concentrated to small volume (about 2–3 mL) under reduced pressure. Then, the solution was added dropwise to 20 mL of acetonitrile, which resulted in an orange/brown precipitate. The mixtures were centrifuged at 3800 rpm for 15 min, precipitates were washed with acetonitrile three times and dissolved in diethyl ether (25 mL) and kept in −20 °C to precipitate the TOAB residue. The reaction mixture was filtered, dried over anhydrous Na_2_SO_4_, and concentrated under reduced pressure. The gold nanoparticles were finally dissolved in CH_2_Cl_2_ and stored in the dark at 4–8 °C. The characteristics of the gold nanoparticles (UV-Vis spectra and TEM image are provided in Supplementary Material (Figure S1)).

### 2.5. Electrode Preparation

**Scheme 2:**
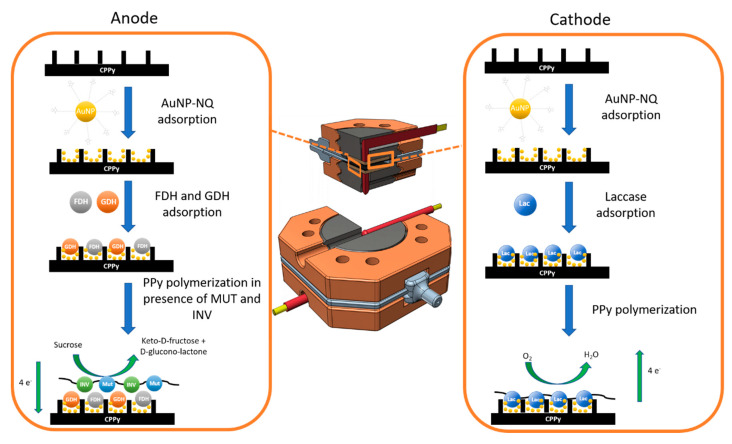
Schematic illustration demonstrating the employed cell and the electrode modification procedures.

The preparation of the electrodes is presented in Scheme 2. To prepare the anode as well as the cathode, AuNP-NQ solution was drop-casted and left to dry on the CPPy electrodes (Figure S2). To prepare the anode, a solution of 1 mg mL^−1^ FDH and GDH was placed on the electrode and incubated for 12 h at 4 °C. Subsequently, the electrodes were immersed in a solution containing INV and MUT (10 mg mL^−1^ in ultrapure water) for 24 h, after which a 2 μL sample of pyrrole monomer was added to the solution. A platinum wire and Ag/AgCl (KCl 3M) electrodes were then immersed in the cell as counter and reference electrodes, respectively. The PPy electrodeposition was carried out using a square wave potential program: 10 s at 0.9 V, followed by 3 s at 0 V for 180 cycles [28]. The optimized thickness of the PPy layer was estimated to be ca. 30 nm. The electrodeposition of the polypyrrole (PPy) layer on the electrodes minimized the leakage of the enzymes. The electrode was then removed from the cell, washed with ultrapure water and stored in the fridge at 4 °C until use. The cathode was prepared in the same way by drop-casting the LAC solution (10 mg mL^−1^) on the CPPy-AuNP-NQ modified electrode and incubation for 12 h, prior to the PPy electropolymerization step. This approach avoids enzyme depletion while small molecules, i.e., organic fuels and oxygen diffusion are hindered to a small extent. Interestingly, the non-redox enzymes, INV and MUT, at the anode preserve most of their native activity. Colorimetric assay of activity showed that immobilization of INV leads to a decrease in its activity from 380 to ca. 180 units mg^−1^.

The fuel cell device consisted of circular CPPy electrodes with a diameter of 1 cm and a mass of about 30 mg. A flow-through device for electrode testing was manufactured using three-dimensional (3D) printing fused filament fabrication (FFF) technology, employing an FFF 3D Printer model Dreamer (Flashforge, Zhejiang, China) from Sygnis New Technologies (Warsaw, Poland). Rigid, plastic parts were printed using PLA (polylactide) filament manufactured by OrbiTech (Jena, Germany). The overall dimensions of the flow vessel are 50 mm (length) × 50 mm (width) × 18 mm (height) and the vessel consists of three main components which were designed to allow rapid disassembling and assembling of the device when replacing the paper electrodes. The middle part of the flow vessel is equipped with channels (with an inner diameter of 1 mm) that ensure the liquid transport into the inner chamber which has an inner diameter of 20 mm, height of 2 mm and a total volume ca. 0.600 mL. The paper electrodes, which were placed on the upper and lower surfaces of the flow chamber as illustrated in Scheme 2, were connected with the potentiostat by steel disc current collectors and tightened with threaded, external components. For all experiments made under flow conditions, flow rate was 1 mL min^−1^.

### 2.6. Experimental Techniques

Homogeneous kinetic experiments were carried out using a quartz cuvette (optical path length −1 cm) at 25 °C with UV-Vis Spectrophotometer UV-2401PC from Shimadzu (Kyoto, Japan). The electrochemical tests were performed using a CH 650d potentiostat (CH instruments, INC., Bee Cave, TX, Austin) and a 0.1 M McIlvaine buffer, pH 6. All long-time tests were made under the constant flow of electrolyte solution. The stability of the devices was tested using galvanostatic charge/discharge applying current density of 10 mA cm^−2^, which was close to maximum current density generated by the fuel cell, within a cell voltage window ranging from 0 to 0.8 V.

The catalytic performances of the bioelectrodes were evaluated by recording voltammograms in a three-electrodes arrangement using a Ag/AgCl (KCl_sat._) reference electrode, a platinum foil counter electrode and the prepared CPPy electrodes as the working electrodes.

The power of the fuel cell was measured in the three-electrode arrangement using a programmable decade resistor. The device enabled the monitoring of the power of the cell under different applied resistances, 0.1 to 10 MΩ, in parallel with measuring the potential of each of the electrodes vs. Ag/AgCl (KCl_sat._) reference electrode during the enzymatic fuel cell operation. Each point in the power plot was measured when the voltage attained a stable value in time. The generated current and power were calculated using Ohm’s law.

## 3. Results

### 3.1. Characterization of the Anode and the Catalytic Process

The electron transfer pathway at the anode involves naphthoquinone as the redox mediator (mediated electron transfer, MET) covalently bound to gold nanoparticles and capped by the FDH or FAD-dependent GDH. Although both enzymes can also be used in the direct electron transfer (DET) mode [29,30,31,32], the use of a naphthoquinone derivative as the mediator was previously reported to be advantageous for these enzymes [33,34] since it leads to a decrease in the overpotential of the catalytic reaction by changing the reaction mechanism: the natural final exit sites of both enzyme-heme subunits are replaced by the mediator moiety characterized by a more negative formal potential [35].

**Figure 1:**
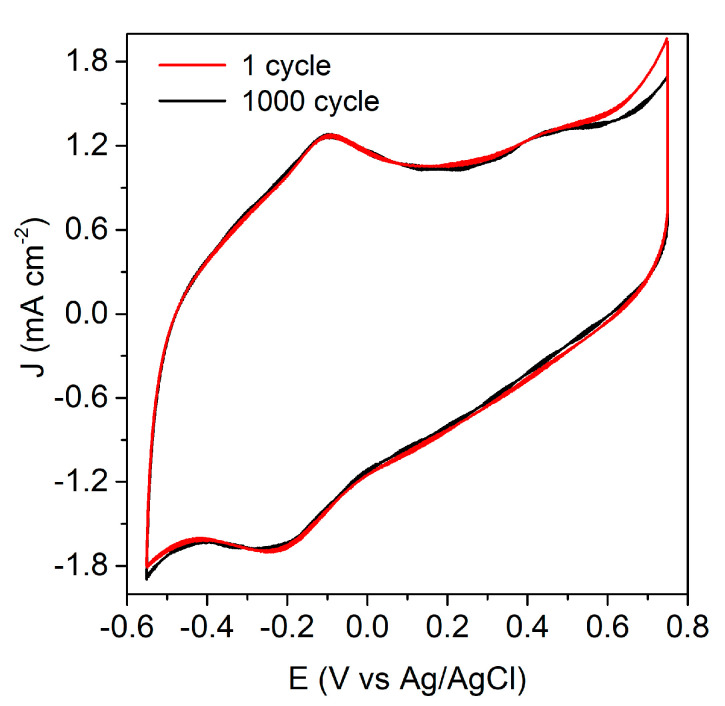
Cyclic voltammograms (showing the 1st and 1000th cycles) recorded for CPPy-electrodes modified with AuNP-NQ in a McIlvaine buffer, pH 5.5 at a scan rate of 1 mV s^−1^.

To achieve efficient MET, the relay molecules were placed at the CPPy electrodes in form of naphthoquinone modified gold nanoparticles, AuNP-NQ [36]. To investigate the stability of this arrangements, we recorded cyclic voltammetry curves on three electrodes and compared the voltammograms of the first and 1000th cycle (Figure 1). No significant decrease in the initial charge under the redox peak of naphthoquinone was seen following cycling, indicating a high durability of electrode material. Subsequently, a layer of pyrrole was electropolymerized at the electrode with INV and MUT present in the reaction mixture. This resulted in the formation of a membrane-like structure with INV and MUT entrapped inside the PPy layer, enabling free diffusion of reagents inside the film [37].

Three different sugars: glucose, fructose, and sucrose may be utilized as the substrates of the bioelectrocatalytic processes at the anode. Sucrose is first hydrolyzed by INV to yield both fructose and α-D-glucose (Equation (1)).

(1) $$\mathrm{sucrose}+H_{2}O\;\overset{\mathrm{INV}}{\Leftrightarrow }\alpha -D-\mathrm{glucose}+\beta -D-\mathrm{fructose}$$

Fructose obtained in this reaction can be oxidized by FDH immediately, whereas the α-D-glucose requires isomerization to β-D-glucose before it can be oxidized by GDH. This isomerization process is catalyzed by the fourth enzyme incorporated in the cascade, MUT:

(2) $$\alpha -D-\mathrm{glucose}\overset{\mathrm{MUT}}{\Leftrightarrow }\beta -D-\mathrm{glucose}$$

Fructose and glucose are oxidized via naphthoquinone-mediated FDH and GDH processes according to mechanisms:

(3) $$\begin{array}{c} \beta -D-\mathrm{glucose}+\mathrm{GDH}\left(\mathrm{FAD}\right)\overset{\;}{\Rightarrow }D-\mathrm{glucono}-1,5-\mathrm{lactone}+\mathrm{GDH}\left({\mathrm{FADH}}_{2}\right)\\ \mathrm{GDH}\left({\mathrm{FADH}}_{2}\right)+\mathrm{NQ}\overset{\;}{\Rightarrow }\mathrm{GDH}\left(\mathrm{FAD}\right)+{\mathrm{NQH}}_{2}\\ {\mathrm{NQH}}_{2}\overset{\;}{\Rightarrow }\mathrm{NQ}+2e^{-}+2H^{+} \end{array}$$

(4) $$\begin{array}{c} \beta -D-\mathrm{fructose}+\mathrm{FDH}\left(\mathrm{FAD}\right)\overset{\;}{\Rightarrow }5-\mathrm{dehydro}-D-\mathrm{fructose}+\mathrm{FDH}\left({\mathrm{FADH}}_{2}\right)\\ \mathrm{FDH}\left({\mathrm{FADH}}_{2}\right)+\mathrm{NQ}\overset{\;}{\Rightarrow }\mathrm{FDH}\left(\mathrm{FAD}\right)+{\mathrm{NQH}}_{2}\\ {\mathrm{NQH}}_{2}\overset{\;}{\Rightarrow }\mathrm{NQ}+2e^{-}+2H^{+} \end{array}$$

**Figure 2:**
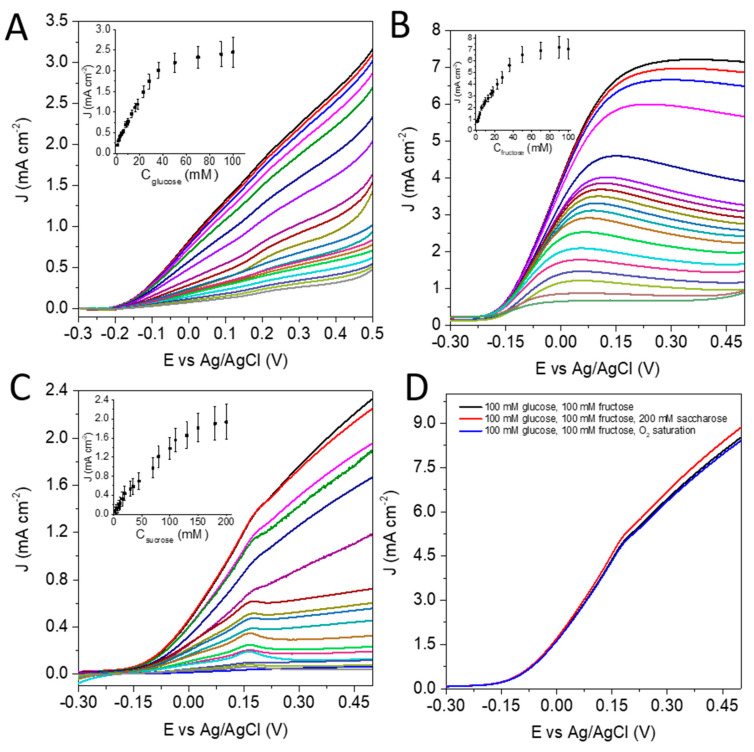
(**A**) Voltammograms demonstrating bioelectrocatalytic activity obtained following the exposure of the invertase (INV)/ mutarotase (MUT)/ glucose dehydrogenase (GDH)/ d-fructose dehydrogenase (FDH) cascade on the anode to solutions containing variable concentrations of glucose, inset: calibration curve based on the currents at E = 0.3 V vs. Ag/AgCl for the different glucose concentrations; (**B**,**C**) the same experiments for fructose and sucrose, respectively, (**D**) LSV responses for INV/MUT/GDH/FDH in substrate mixtures. All measurements were performed at a scan rate of 5 mV s^−1^. Unless otherwise indicated, prior to each measurement the electrolyte (McIlvaine buffer pH 6) was purged with argon. All curves are shown after background subtraction. The standard deviation error bars were derived based on five experiments.

Figure 2A–C show linear sweep voltammograms demonstrating the catalytic activity of the INV/MUT/GDH/FDH cascade immobilized on the CPPy electrode with AuNP-NQ as the mediator for different concentrations of glucose, fructose and sucrose. The onset of the bioelectrocatalytic current appears at −0.22 V vs. Ag/AgCl. The half-wave potential for fructose oxidation is close to the one of naphthoquinone covalently bound to AuNPs (−0.13 V vs. Ag/AgCl, Figure 1).

**Figure 3:**
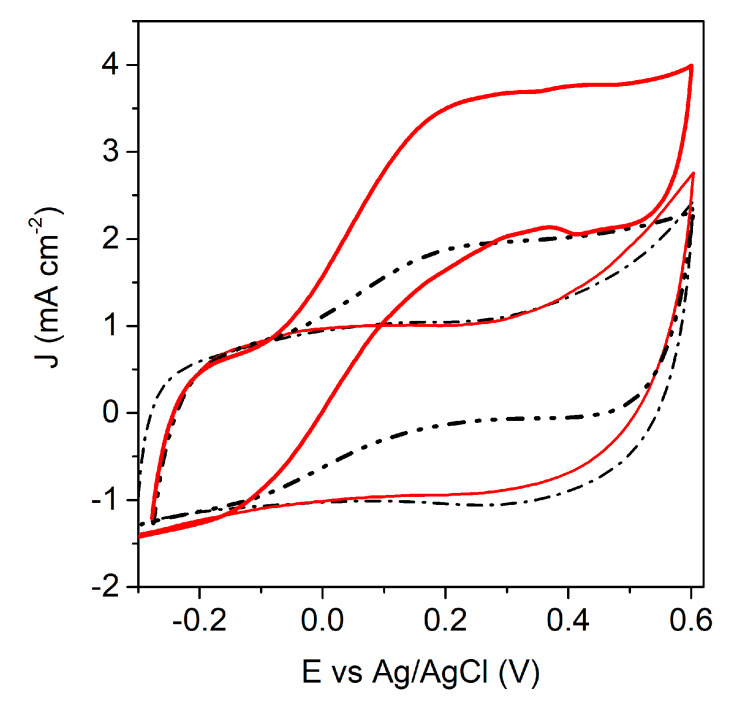
Cyclic voltammogram of cellulose/polypyrrole (CPPy)/INV/MUT/FDH/GDH but without AuNP-NQ on the electrode in the presence of 100 mM fructose (red solid line) and 100 mM glucose (black dashed line); all measurements were performed at a scan rate of 5 mV s^−1^. McIlvaine buffer pH 6 was purged with argon. Thinner lines denote background currents in the absence of sugar.

In case of glucose oxidation by GDH and products of sucrose hydrolysis it is rather difficult to determine the half-wave potential value since the limiting current is not well developed. In the presence of AuNP-NQ, the electron transfer mechanism switches from MET to DET. For comparison, the catalytic activity of INV/MUT/GDH/FDH cascade without AuNP-NQ is shown in Figure 3. The overpotential of the catalytic reaction is significantly increased—the onset of current appears at ca. −0.1 V vs. Ag/AgCl and the obtained limiting currents in 100 mM glucose or fructose solutions are reduced ca. twice.

The catalytic currents increase with increasing concentrations of the fuels (see the insets in Figure 2A–C). These results indicate that the multienzyme assembly used to modify the anode indeed supports the oxidation of these three fuels by providing an anodic pathway for the cascade reactions with kinetics that are characteristic for the enzymes. It is, however, worth mentioning that the Michaelis–Menten constants calculated from calibration curves for glucose and fructose oxidation are significantly larger than those reported in the literature. Hindered affinity of the substrate to the active site might be attributed to the overall complexity of the system.

**Figure 4:**
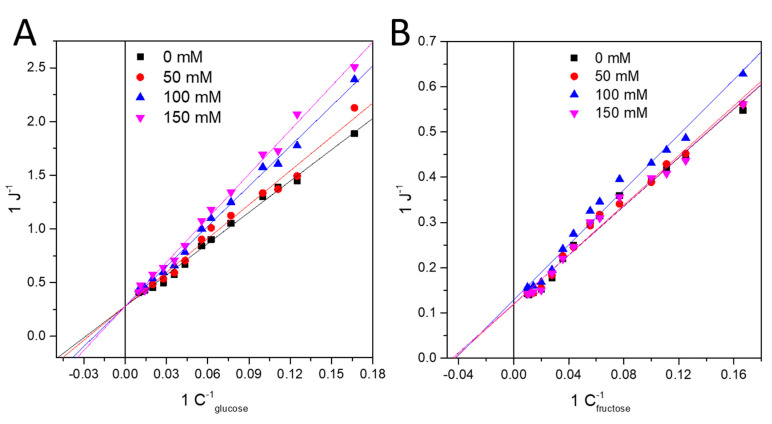
Lineweaver–Burk plots obtained using data extracted from voltammetric experiments for the anode containing one oxidoreductase: GDH in the presence of increasing concentrations of fructose (**A**), and FDH in the presence of an increasing concentrations of glucose (**B**).

The currents obtained for the solution of fructose are larger than those for glucose or sucrose. Here, it should be noted that the substrate concentrations needed to reach the limiting values of current are significantly higher for sucrose, 200 mM, than for glucose or fructose (100 mM). This indicates that the prior pretreatment with INV and MUT significantly influence the kinetics of enzymatic cascade. Another factor affecting the catalytic activity can be the competitive inhibition of the oxidoreductases collected on the anode. Lineweaver–Burk plots obtained for electrodes with only one oxidoreductase: FDH or GDH, are shown in Figure 4, for different concentrations of the competing monosaccharides. While the presence of glucose in the reaction solution does not affect the FDH kinetics (no significant change of K_M_ was observed), the presence of fructose causes an evident change of the K_M_ for the GDH while J_max_ stays the same. It suggests contribution of competitive inhibition with an inhibition coefficient K_i_ of approximately 0.55. As mentioned earlier, the native activities of INV and MUT are decreased upon immobilization in the PPy matrix, which decreases in turn the rate of generation of fructose and glucose. Whereas one would expect to have a full conversion of each sucrose reactant into fructose and glucose molecules, a significant fraction of these molecules is most likely lost, due to their diffusion to the electrolyte before they can be oxidized. It means that only a fraction of products of INV and MUT reactions undergo reaction with oxidoreductases.

To evaluate the additivity of the processes of different fuels, a set of voltammograms was recorded (Figure 2D). The bioelectrocatalytic current obtained upon exposing the INV/MUT/GDH/FDH-cascade anode to a buffer solution containing all three sugars at concentrations yielding the largest limiting currents is ca. 8.1 mA cm^−2^ and exceeds the saturation currents observed for the separately introduced fuels. The limiting current is, however, smaller (89%) than the current obtained by summing up the currents recorded for individual sugars. The observed minor losses may be due to increased viscosity of electrolyte in the presence of all sugars and hence lower diffusion coefficients of the substrates. Adding sucrose did not drastically affect the observed current density output. No significant differences in the current densities were seen in the presence and absence of dioxygen reflecting that the enzymes are not sensitive towards oxygen.

In Table 1, contributions of the different enzymes to the overall anodic response are shown. These experiments were carried out using 200 mM sucrose solution and the electrocatalytic currents were measured at 0.3 V vs. Ag/AgCl. By eliminating the first cascade step-sucrose hydrolysis, FDH and GDH are deprived of their substrates and the current is very low. The residual current observed without INV could be connected to the spontaneous slow hydrolysis of sucrose [38]. With INV but without MUT, the total current density, including the contribution from the hydrolyzed sucrose, reached about 1.11 mA cm^−2^. It was expected, considering the contribution of the INV-catalyzed hydrolysis of sucrose to its products, fructose and glucose, with the latter also naturally equilibrating between its α and β isomer forms (though at a slower rate in the absence of MUT). The current responses also suggest that the transformations in the presence of INV and MUT were roughly in accordance with a stoichiometric sucrose transformation to fructose and glucose. These results highlight the contribution of each of the cascade steps and demonstrate the need for all four enzymes in order to amplify the overall biolectrocatalytic performance of the anode.

### 3.2. Characterization of the Biocathode and the Catalytic Process

As the enzymatic cascade at the anode responded effectively to the presence of the different fuels and was not sensitive to O_2_, it was assumed that the anode could be coupled with the enzymatic cathode composed of AuNP-NQ and LAC deposited on CPPy electrode (Scheme 2).

**Figure 5:**
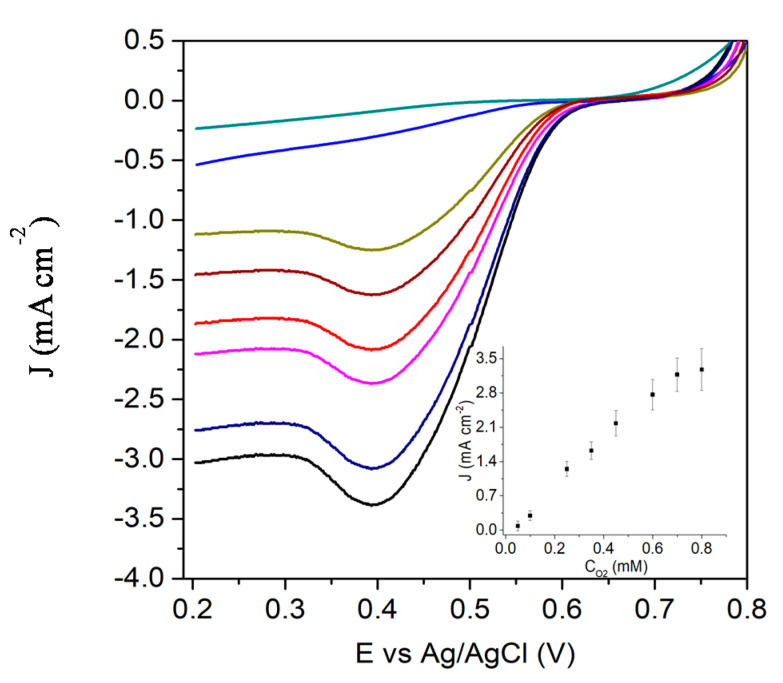
Voltammograms demonstrating the bioelectrocatalytic activity when laccase (LAC) cathode is exposed to solutions of increasing O_2_ concentrations. Inset: Current density measured at 0.25 V vs. Ag/AgCl for the different O_2_ concentrations. Scan rate: 5 mV s^−1^. All curves were background corrected. The standard deviation error bars based on 5 experiments.

Laccase is known for its relatively high stability while functioning as an enzymatic catalyst for 4e^−^ reduction of O_2_ to water. However, to provide efficient electron transfer from the electrode to the entrance active site of LAC–T1 Cu site, the presence of a mediator or orienting agent, enabling electron transport, is required. In our previous report, we showed that LAC immobilized on the surface of CPPy can transfer electrons both according to MET and DET mechanisms [37]. However, based on the affinity of naphthoquinone to the hydrophobic T1 site [39,40], more efficient electron contact can be achieved resulting in significant increase in current density. Indeed, the current density reaches ca. 3 mA cm^−2^ in O_2_ saturated solution (Figure 5). The Michaelis–Menten constant, K_M_, was 0.36 mM suggesting that the affinity of the substrate to the active site is decreased. It may be explained by the presence of the electrodeposited PPy layer used to minimize the enzyme desorption.

### 3.3. The Charge Generation Properties of the EFC

**Figure 6:**
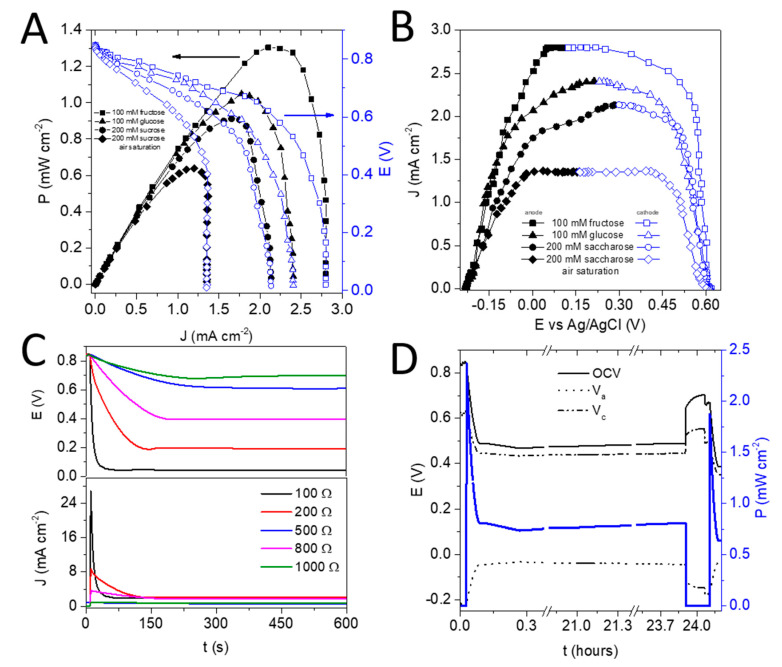
(**A**) Polarization (blue lines) and power (black line) curves for the enzymatic fuel cells (EFC) containing the enzymatic cascade anode and the LAC cathode in O_2_ or air saturated solutions of different fuels; (**B**) the current generated as a function of anode and cathode potentials recorded in O_2_ saturated solutions of different fuels; (**C**) voltage and current density of the device vs. operation time under different resistances applied; (**D**) stability experiment under 600 Ω resistance during a 24 h test; experiments C and D were performed in O_2_ saturated 200 mM sucrose solution, under constant flow conditions.

The anode and the cathode were placed in the constructed fuel cell and its performance was studied under different external resistances. Figure 6A shows polarization and power curves recorded in a 200 mM sucrose solution saturated with O_2_ or air. The open circuit voltage (OCV) for the device was 0.84 ± 0.04 V, close to the value expected based on the onset potentials of the voltammetric currents presented in the previous section. The average maximum current density J_max_ and power density P_max_ strongly depended on the type of fuel mixture utilized in the experiment. For O_2_-saturated 100 mM fructose solution J_max_ at short circuit was 2.8 mA cm^−2^ (Figure 6A) and P_max_ was 1.3 mW cm^−2^. Figure 6B shows changes of electrode potentials vs. reference electrodes during the work of the fuel cell where the cathode potential is shifted to more negative values, suggesting that the reduction process at the cathode limits, in this case, the fuel cell performance. The plateau regions of the voltage drop connected to the ohmic resistances were clearly comparable (down to approximately 0.5 V), when maximum current was generated. For lower voltages, the observed current density does not change, therefore it can be assumed that the differences in the cathode performance are caused by mass transfer limitations of oxygen. Further results correlate well with CV data described earlier—in the presence of 100 mM glucose J_max_ and P_max_ were 2.4 mA cm^−2^ and 1.1 mW cm^−2^, respectively, in 200 mM sucrose solution −1.7 mA cm^−2^ and 0.81 mW cm^−2^. When the electrolyte was saturated with air, these parameters dropped to 1.3 mA cm^−2^ and 0.63 mW cm^−2^. A comparative experiment performed in the absence of substrates proved that the obtained voltage and power values are due to the electrocatalytic reaction since only residual currents remained, possibly due to differences of active sites potentials (OCV 0.2 V, P_max_ 0.023 mW cm^−2^) (Figure S3).

Figure 6C depicts discharging profiles under different external resistances. Crucial for EFC with supercapacitor properties is that the discharge time is relatively long for each load. It must be pointed out that the observed discharge profiles are superposition of the release of the gathered charge and the charge generated in the bioelectrocatalytic reactions.

In the time stability tests, 5 kΩ resistance was applied to the circuit for 24 h (Figure 6D). During the first 2 min, the observed power was significantly higher (2.3 mW cm^−2^) due to internal capacitance. At longer times, the power became constant, ca. 0.81 mW cm^−2^, confirming good stability of the system in time. Interestingly, the voltage of the cell and of the individual electrodes slightly increased during the 24-h test. This confirmed that during discharging under external resistance both electrodes are also partially loaded due to their capacitive properties. When the measurement was switched almost off, i.e., by applying resistance of 0.1 MΩ, the voltage of the cell increased to almost OCV value (ca. 0.72 V). Upon repeating the experiment, again, a similar power spike initially appeared and was followed by a decrease in power to almost constant value at longer times. In Table 2, we compare the parameters of the biofuel cell constructed in this study with the enzymatic cascade-based fuel cells reported in the literature.

## 4. Conclusions

A sucrose/air paper-based enzymatic fuel cell has been constructed with an enzymatic cascade, composed of invertase, mutarotase, FAD-dependent glucose dehydrogenase and fructose dehydrogenase on the anode and the laccase-modified cathode. The combination of four enzymes enabled efficient transformation of sucrose into β-glucose and β-fructose, and their utilization as the fuel. The naphthoquinone modified gold nanoparticles, immobilized on the paper-based anode, enable mediated electron transfer from the anodic enzymes at a decreased overpotential. They also lead to favorable orientation of laccase molecules at the cathode due to hydrophobic interaction of naphthoquinone with the T_1_ pocket. The power density of the constructed fuel cell was 0.81 mW cm^−2^ and the OCV was 0.82 V. Highly favorable properties, both in terms of power and OCV, compared to those of other biofuel cells [16,23,44], and in particular compared to the sucrose-based system described by Herkendell et al. [42] which is the result of the innovative procedure used for the electrode preparation (Table 2). The CPPy-AuNP-NQ modification provides not only good catalytic but also capacitive properties of both anode and cathode. The power density, although advantageous, is limited by the efficiency of the enzymatic cascade on the anode and mass transport of the substrates to the electrode surfaces. It should be mentioned that during long term discharging under external resistance, both electrodes were also loaded to some extent, and when the measurement was switched almost off, i.e., under 0.1 MΩ, the voltage of the cell increased to almost OCV value (ca. 0.72 V) revealing the contribution of the self-regeneration process.

Significant stability of the system should be underlined—approximately 36% of activity was retained after 8 days. All these properties place the described system among the most efficient cascade fuel cells reported so far.

## Figures and Tables

**Table 1:** Current densities measured at +0.3 V for different combinations of enzymes on the anode in the presence of 200 mM sucrose solution.

INV	X	X	X	X		X			X		X	X			
**MUT**	X	X	X		X	X	X			X			X		
**GDH**	X	X		X	X		X	X			X			X	
**FDH**	X		X	X	X			X	X	X					X
**J (mA cm^−2^)**	1.93 ± 0.37	0.77 ± 0.18	1.03 ± 0.18	1.11 ± 0.25	0.05 ± 0.01	0.0 ± 0.0	0.04 ± 0.01	0.06 ± 0.01	1.01 ± 0.23	0.03 ± 0.01	0.48 ± 0.06	0.0 ± 0.0	0.0 ± 0.0	0.02 ± 0.01	0.03 ± 0.01

**X**—in the presence of respective enzyme.

**Table 2:** Comparison of enzymatic cascade-based fuel cells for sucrose oxidation.

Enzymes	Electrode Materials	Fuel Solution	OCV (V)	P_max_ (µW cm^−2^)	J_max_ (µA cm^−2^)	Stability	Ref.
INV, FDH, glucose oxidase	Anode: Toray Paper ferrocene-based hydrogel; Cathode: Pt electrode	100 mM sucrose; O_2_ saturated	0.61	50	340		[5]
INV, glucose oxidase	Anode: carbon paper, DNA template prepared by rolling circle amplification; Cathode: Pt electrode	50 mM sucrose; O_2_ saturated	0.59	22.2	140		[41]
INV, MUT, FDH, glucose oxidase LAC, catalase	Mesoporous carbon nanoparticle matrix	100 mM glucose, 100 mM fructose, 180 mM sucrose, O_2_ saturated, 140 mM H_2_O_2_	0.64	240	890		[42]
sucrose phosphorylase, xylose isomerase, polyphosphate glucokinase, glucose-6-phosphate DH, 6-phosphogluconate DH, PPP enzymes	Carbon paper, enzymes and mediator dissolved in anolyte; Cathode: Pt electrode	0.64 mM sucrose, O_3_ saturated	0.80	690	1330	27% of initial power after 6 days	[43]
INV, MUT, FDH, GDH; LAC	CPPy, AuNQ modified with naphthoquinone	200 mM sucrose; O_2_ saturated	0.82	810	2230	36% of initial power after 8 days	This paper

## Supplementary Materials

The following are available online at https://www.mdpi.com/2079-4991/10/8/1534/s1, Figure S1: Characteristics of AuNP-NQ; Figure S2: SEM image CPPy paper; Figure S3: Polarization (blue lines) and power (black line) curve for the full fuel cell containing an enzymatic cascade anode and a LAC cathode in deoxygenated McIlvaine buffer pH 5.
