# Supplementary material for: Multi-Substrate Biofuel Cell Utilizing Glucose, Fructose and Sucrose as the Anode Fuels
Source: Nanomaterials (Basel). 2020 Aug 5;10(8):1534. doi: 10.3390/nano10081534 (PMC7466598; doi:10.3390/nano10081534)
Supplement: Supplementary file 1 [file nanomaterials-10-01534-s001.pdf]

# Supplementary Materials: Multi-Substrate Biofuel Cell Utilizing Glucose, Fructose and Sucrose as the Anode Fuels †

**Michał Kizling, Maciej Dzwonek, Anna Nowak, Łukasz Tymecki, Krzysztof Stolarczyk, Agnieszka Więckowska and Renata Bilewicz ***

Faculty of Chemistry, University of Warsaw, 1 Pasteura Str., 02-093 Warsaw, Poland; mkizling@gmail.com (M.K.); maciejdzwonek@student.uw.edu.pl (M.D.); a.nowak@uw.edu.pl (A.N.); luktym@chem.uw.edu.pl (L.T.); kstolar@chem.uw.edu.pl (K.S.); awiec@chem.uw.edu.pl (A.W.)

* Correspondence: bilewicz@chem.uw.edu.pl; Tel.: +48-22-55-26-357

† This paper is a part of Ph.D. Thesis of Michal Kizling, presented at the University of Warsaw (Poland).

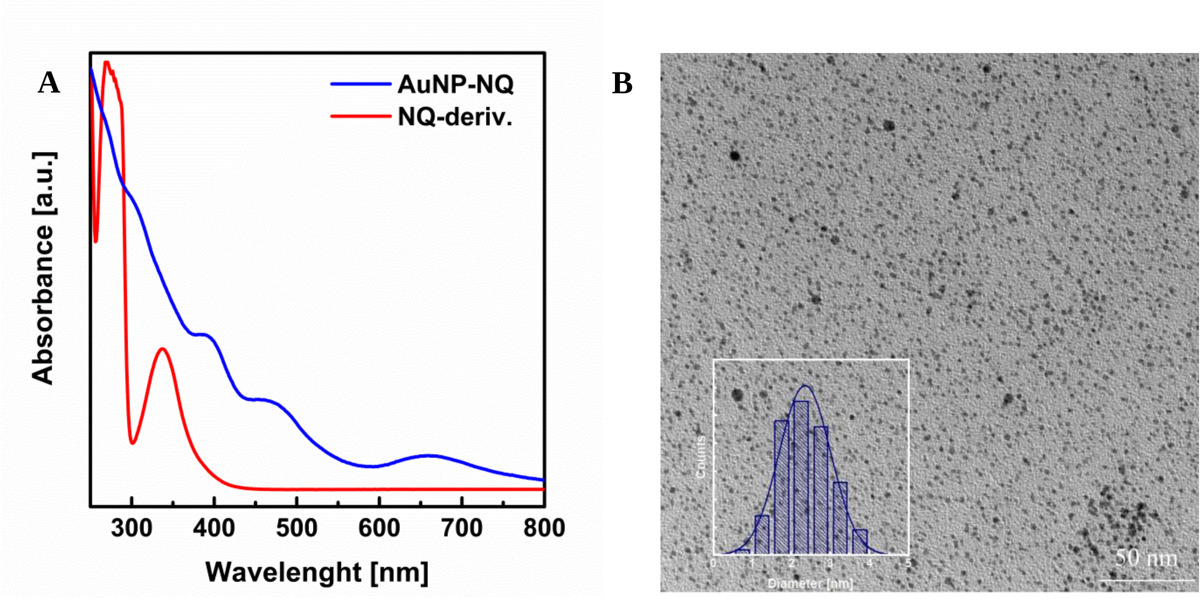


**Figure S1.** Characteristics of AuNP-NQ: A) UV-Vis spectra (red – NQ derivative, blue – AuNP modified with synthesized NQ-derivative) all measured in $CH_2Cl_2$; and B) TEM image (mean diameter 2.3 ± 0.6 nm), scale bar – 50 nm.

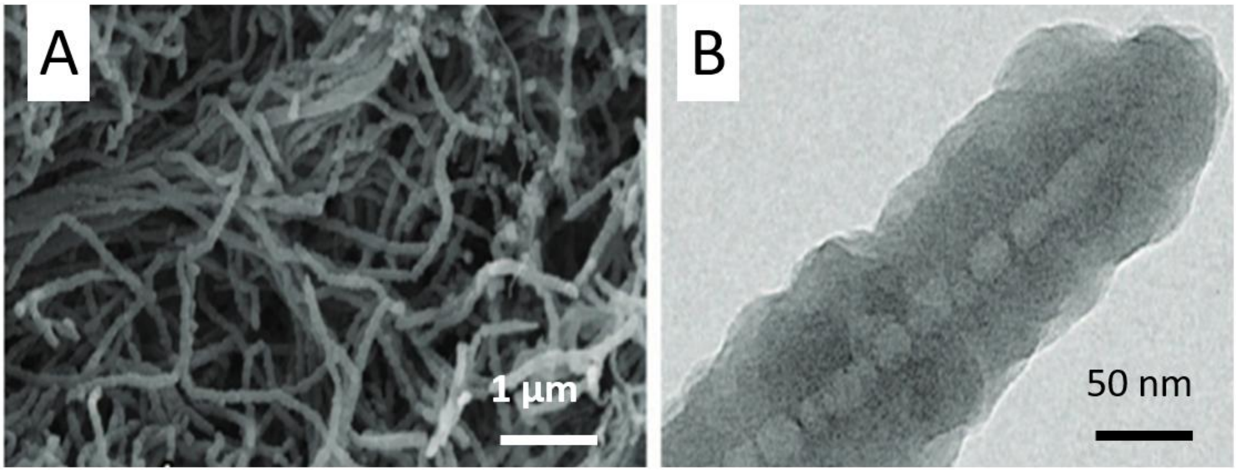


**Figure S2.** SEM image CPPy paper; B) TEM image of separated CPPy fibre

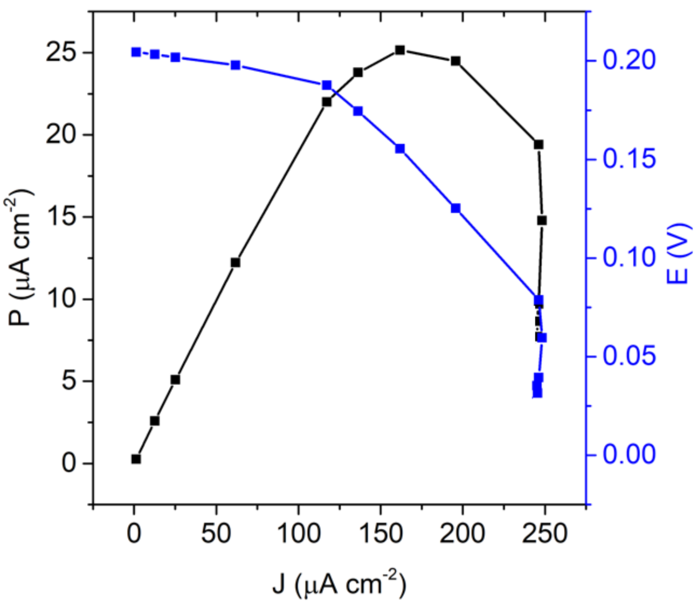


**Figure S3** Polarization (blue lines) and power (black line) curve for the full fuel cell containing an enzymatic cascade anode and a LAC cathode in deoxygenated McIlvaine buffer pH 5
